# Vitamin D and Calcium Are Required during Denosumab Treatment in Osteoporosis with Rheumatoid Arthritis

**DOI:** 10.3390/nu9050428

**Published:** 2017-04-26

**Authors:** Yukio Nakamura, Takako Suzuki, Tomohiko Yoshida, Hideshi Yamazaki, Hiroyuki Kato

**Affiliations:** 1 Department of Orthopaedic Surgery, Shinshu University School of Medicine, Asahi 3-1-1, Matsumoto 390-8621, Japan; takako1119@shinshu-u.ac.jp (T.S.); hirokato@shinshu-u.ac.jp (H.K.); 2 Department of Rheumatology, Toshin Yoshida Internal Medicine, Gomyo 643-2, Sakaki-Machi 389-0606, Japan; yoshidatomohiko@me.com; 3 Department of Rheumatology, Marunouchi Hospital, Nagisa 1-7-45, Matsumoto 390-8601, Japan; yamazaki_h@marunouchi.or.jp

**Keywords:** calcium, denosumab, osteoporosis, rheumatoid arthritis, vitamin D

## Abstract

The aim of this 12-month retrospective study was to evaluate differences in the outcomes of denosumab alone or denosumab combined with vitamin D and calcium supplementation in patients having osteoporosis (OP) with rheumatoid arthritis (RA). Patients were divided into the denosumab monotherapy group (denosumab group, 22 cases) or denosumab plus vitamin D supplementation group (combination group, 21 cases). We measured serum bone alkaline phosphatase (BAP), N-terminal propeptide of type 1 collagen (P1NP), tartrate-resistant acid phosphatase (TRACP)-5b, and urinary N-terminal telopeptide of type-I collagen (NTX) at baseline, 1 week, and 1, 2, 4, 6, 8, and 12 months. We also assessed bone mineral density (BMD) of the lumbar 1-4 vertebrae (L-BMD) and bilateral total hips (H-BMD) at baseline and 4, 8, and 12 months. Matrix metalloprotanase-3 (MMP-3), Disease Activity Score-28 C-reactive protein (DAS28-CRP), Simplified Disease Activity Index (SDAI), and Health Assessment Questionnaire-Disability Index (HAQ-DI) were assessed before treatment and at 12 months to evaluate RA conditions. The study results showed that BAP, TRACP-5b, and NTX were significantly decreased, but tended to return to pre-treatment levels around 6 and 12 months in both groups. While L-BMD and H-BMD substantially increased in both groups, H-BMD had become significantly higher in the combination group at 12 months (*p* < 0.01) as compared with the denosumab group. There were no significant differences between the groups regarding MMP-3, DAS28-CRP, SDAI, or HAQ-DI. Compared with denosumab monotherapy, combination therapy of denosumab with vitamin D and calcium significantly increased H-BMD in patients having OP with RA.

## 1. Introduction

Rheumatoid arthritis (RA) is characterized by chronic synovitis and receptor activator of nuclear factor-ligand (RANKL)-dependent osteoclastogenesis leading to bone damage and ensuing severe physical disability. Focal and systemic joint destruction, including secondary osteoporosis (OP), contributes prominently to the morbidity associated with RA.

Current therapies for primary and secondary OP are based on our understanding of bone biology. Denosumab, a fully human monoclonal antibody against RANKL, has been shown to selectively inhibit osteoclastogenesis. Consequently, the drug strongly abrogates bone resorption, increases bone mineral density (BMD), and prevents fragility fractures [1]. The very recent FREEDOM open-label extension study also demonstrated that nonvertebral fracture rate was decreased for up to 10 years after denosumab treatment and that BMD values increased linearly [2]. Thus, denosumab is a good therapeutic option for OP patients with respect to the increase of BMD, improvement in bone turnover markers, and prevention of fractures.

The frequency of OP in patients with RA is approximately 15% according to the Kvien et al. [3] and Arain reported low BMD in a quarter of patients in the earlier stages of RA [4]. OP is therefore a major complication of RA. As the occurrence of femoral neck fractures and vertebral compression fractures in individuals with RA is twice as high as that in healthy individuals of the same age [5], appropriate OP drugs are considered to be essential for fracture prevention in RA patients.

In 1999, the Japanese Ministry of Health published *Guidelines for the prevention and treatment of osteoporosis*, which stated that whenever a placebo group was used as a control group against a drug group, sufficient calcium and vitamin D should be administered as baseline treatment [6]. Hence, calcium addition and vitamin D supplementation are routinely used in studies carried out in Japan.

With respect to the metabolism of vitamin D, “active” vitamin D, such as calcitriol (1,25-dihidroxycholecalciferol;1-25(OH)_2_D), regulates calcium metabolism [7]. “Native” vitamin D is cholecalciferol. The latter is hydroxylated in the liver to become 25(OH)D_3_ (calcifediol), which is then hydroxylated in the kidneys to become active vitamin D [8]. The type of vitamin D commonly adopted in research studies is native vitamin D. In a Japanese phase-III study ofalendronate, calcium (600 mg) and native vitamin D (400 IU) were used [9]. Moreover, after the approval of denosumab use, calcium and vitamin D have been recommended to prevent hypocalcemia in OP treatment using denosumab. Denotas^®^ Chewable (vitamin D and calcium supplementation) has accordingly become approved for denosumab treatment.

Hypocalcemia is considered to be one of the most common adverse effects in denosumab treatment for OP [10,11]. However, the mechanism by which hypocalcemia occurs, which may or may not include vitamin D, is unknown. Okada reported that denosumab could cause hypocalcemia [12]. Others have advised that calcium and vitamin D be taken together during denosumab administration in OP treatment [13,14]. We investigated if supplementation with vitamin D had additive effects on markers of bone metabolism and BMD in Japanese OPpatients with RA.

The aim of this 12-month retrospective study was to evaluate differences in the outcomes of denosumab alone or denosumab combined with vitamin D and calcium supplementation in OP patients with RA.

## 2. Materials and Methods

The inclusion criteria of this 12-month retrospective study was OP patients with low bilateral total hip BMD (H-BMD) and/or lumbar 1-4 BMD (L-BMD) (i.e., less than −3.0 standard deviation (SD)) with RA. The exclusion criteria in this study were patients with chronic renal failure (estimated glomerular filtration rate (eGFR) <40 mL/min/1.73 m^2^), bone metabolic disorders or diabetes mellitus, both of which affect OP, and fracture within 1 year prior to the study. The diagnosis of primary OP was made in accordance with the revised criteria established by the Japanese Society of Bone and Mineral Research [15]. The diagnosis and treatment of RA were conducted in accordance with the 2010 American Collage of Rheumatology (ACR)/European League Against Rheumatism (EULAR) classification system [16]. Forty-three Japanese female OP patients having RA with low-to-moderate disease activity (2.6 < disease activity score (DAS) 28 ≤ 5.1) were recruited at our institution between 2014 and 2016 (patient information is summarized in Table 1). The patients were retrospectively classified into the denosumab alone treatment group (denosumab group; 22 cases) or the denosumab plus vitamin D and calcium supplementation treatment group (combination group; 21 cases) matched on the basis of age, gender, body mass index (BMI), pre-treated bisphosphonate (BP) period, disease duration of RA, and disease activity (Table 1). BP treatment was ceased prior to study commencement. Mean age was 70.9 ± 1.8 years in the denosumab group and 70.6 ± 2.3 years in the combination group. All enrolled patients had received BP pre-treatment for an average duration of 5.8 ± 1.0 years in the denosumab group and 5.5 ± 1.0 years in the combination group. The average doses of methotrexate (MTX) and prednisolone (PSL) in the denosumab and combination groups were 6.7 ± 0.75 and 6.6 ± 0.85 mg/day and 4.5 ± 0.5 and 5.0 ± 0.0 mg/day, respectively (Table 1). 

Steinbroker classification immediately prior to denosumab treatment disclosed 4 patients with stage I, 5 with stage II, 4 with stage III, and 9 with stage IV in the denosumab group and 4 patients with stage I, 5 with stage II, 4 with stage III, and 8 with stage IV in the combination group. There were 16 patients with class I and 6 with class II in the denosumab group and 15 patients with class I, 5 with class II, and 1 with class IV in the combination group.

All serologic analyses were conducted just prior to denosumab commencement (baseline) and at 12 months of treatment using cryogenically stored samples by commercially available kits in accordance with each manufacturer’s instructions, including matrix metalloproteinase-3 (MMP-3; Kyowa Pharma Chemicals, Toyama, Japan). We also examined changes in disease activity score (DAS)28-C-reactive protein (CRP), simplified disease activity index (SDAI), and patient-reported health assessment questionnaire-disability index (HAQ-DI) as indicators of RA status for all patients at the same time points. All data are expressed as the mean ±SE.

Each patient received denosumab (60 mg, s.c.) once every 6 months in both groups. In the combination group, we gave newly approved vitamin D supplementation tablets (762.5 mg of precipitated calcium carbonate, 200 IU of cholecalciferol, 59.2 mg of magnesium carbonate) twice daily to all patients after denosumab administration.

Serum bone alkaline phosphatase (BAP) and N-terminal propeptide of type 1 procollagen (P1NP) were measured as bone formation markers using a chemiluminescent enzyme immunoassay. Serum tartrate-resistant acid phosphatase (TRACP)-5b and urinary N-terminal telopeptide of type I collagen (NTX) (Osteomark, Osteox International, Seattle, WA, USA) were assessed as markers of bone resorption. TRACP-5b and urinary NTX were measured using ELISA. Serum levels of whole parathyroid hormone (PTH 1–84) were evaluated as bone turnover markers by immunoradiometric assays. Serum levels of 1,25(OH)_2_D_3_ were measured as a bone turnover marker by immunoradiometric assays. After overnight fasting and omitting the first morning samples, serum and urine were collected between 8:30 a.m. and 11:00 a.m. Immunoassays were performed by SRL Inc. (Tokyo, Japan). Serum samples were stored at −80 °C until bone turnover marker assessment at the end of the study. Samples were collected before treatment administration, at 1 week, and at 1, 2, 4, 6, 8, and 12 months after denosumab treatment.

Bone turnover markers and BMD were determined for each time point and comparisons were made between the groups using statistical analysis. BMD was measured using a dual-energy X-ray absorption (DXA) fan-beam bone densitometer (Lunar Prodigy; GE Healthcare Bio-Sciences Corp., Piscataway, NJ, USA) at the L1–4 levels of the posteroanterior spine and total bilateral hips. Routine quality control was ensured using a phantom box. Fracture sites were avoided during the evaluation of BMD. BMD was examined before treatment administration and at 4, 8, and 12 months. Physicians interpreting BMD assessments and DXA measurements and the laboratory staff performing the bone marker assays were blinded to the treatment groups.

In both groups, the percent changes of markers were determined at each time point using Bonferroni correction for multiple comparisons. Marker comparisons between the groups at each time point were performed by Welch’s *t*-test. *p*-values of <0.01 or <0.05 were considered to be statistically significant. Based on a SD of 2.5% and sample sizes of 22 in the denosumab group and 21 in the combination group, we calculated that the study had 80% power to detect at least a 5% difference in L-BMD.

This investigation was approved by the Institutional Ethical Review Board of Shinshu University School of Medicine (#2365), Japan, prior to its commencement. Written informed consent was obtained from all subjects. Study methods were carried out in accordance with the approved guidelines.

## 3. Results

All 43 enrolled patients attended all scheduled visits over the 1-year observational period (Table 1). Table 2 and Table 3 present the value and percent changes, respectively, of key parameters at the study end point.

### 3.1. Serum Corrected Calcium and Phosphorus (P) Levels

**Figure 1:**
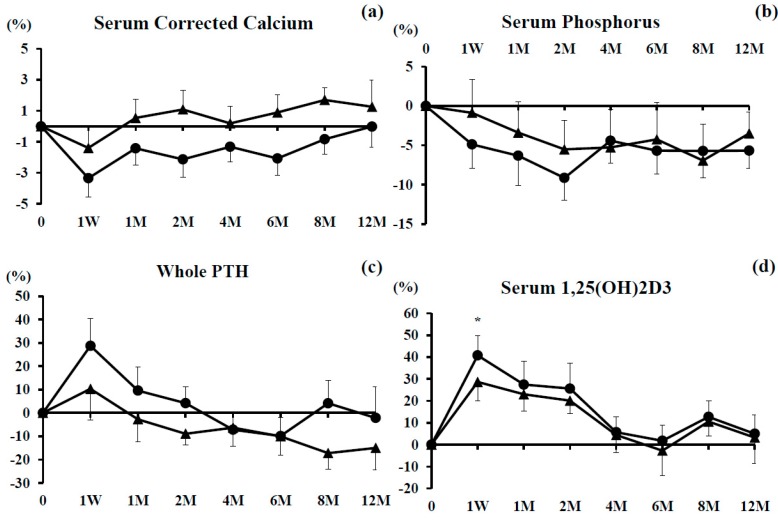
Percent changes of serum calcium (**a**); serum phosphorus (**b**); serum whole parathyroid hormone (PTH) (**c**); serum 1,25(OH)_2_D_3_ (**d**) over the 12-month study period. Closed circles show the denosumab monotherapy group and closed triangles show the combination group. Single asterisk (*) denotes significant differences (*p* < 0.05) at each time point compared with pretreatment in denosumab monotherapy.

The percent changes of serum calcium and P were not significant in either group during the observational period as compared with baseline. Although there were no significant differences in these parameters between the groups, there was a tendency for the percent change of serum calcium in the combination group to be superior to that in the denosumab group (Figure 1a,b). 

### 3.2. Serum Whole PTH and 1,25(OH)_2_D_3_

The percent change of serum whole PTH increased at 1 week and then decreased gradually during the study period in both groups, with no significant differences between them (Figure 1c).

The percent change of serum 1,25(OH)_2_D_3_ was significantly increased at 1 week only in the denosumab monotherapy group. It tended to increase until the first week of observation in both groups, after which it returned to baseline by 6 months, then increased slightly, and gradually decreased from 8–12 months in both groups (Figure 1d).

### 3.3. Bone Turnover Markers

#### 3.3.1. Bone Formation Markers

**Figure 2:**
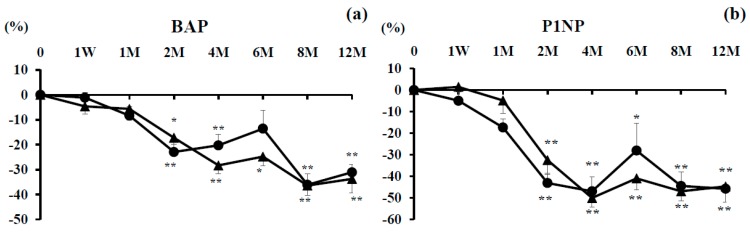

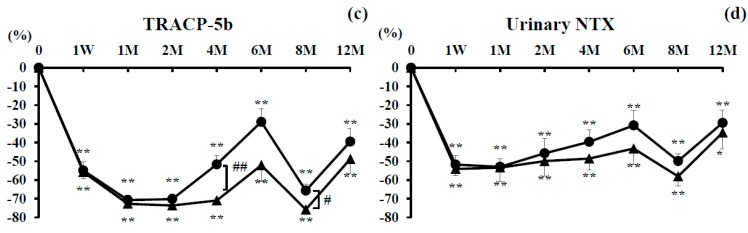
Percent changes of serum bone-specific alkaline phosphatase (BAP) (**a**); serum N-terminal propeptide of type 1 procollagen (P1NP) (**b**); serum tartrate-resistant acid phosphatase (TRACP)-5b (**c**); urinary cross-linked N-terminal telopeptides of type I collagen (NTX) (**d**) over the 12-month study period. Closed circles show the denosumab monotherapy group and closed triangles show the combination group. Single asterisk (*) denotes significant differences (*p* < 0.05) and double asterisks (**) denote significant differences (*p* < 0.01) at each time point compared with pretreatment in the denosumab monotherapy or combination groups. Single hashtag (#) shows significant differences *(p <* 0.05) and double hashtags (##) show significant differences (*p* < 0.01) between the denosumab monotherapy and combination groups at each time point.

The percent change of serum BAP was significantly decreased at 2, 4, 8, and 12 months in the denosumab group and at 2, 4, 6, 8, and 12 months in the combination group. There were no significant differences between them (Figure 2a).

The percent change of serum P1NP tended to decrease from 2 to 12 months in both groups, with no significant difference between the groups (Figure 2b).

#### 3.3.2. Bone Resorption Markers

The percent change of TRACP-5b was significantly decreased at every time point compared with baseline in both groups. There were significant differences at 4 and 8 months between the groups. Decreased TRACP-5b tended to return to baseline at 6 and 12 months of treatment (Figure 2c).

The percent change of urinary NTX was significantly decreased compared with baseline in both groups at every time point. There were no significant differences between the groups. Decreased urinary NTX tended to return to pre-treatment levels after 2 months, with the exception of 8 months (Figure 2d).

#### 3.3.3. L-BMD and H-BMD

**Figure 3:**
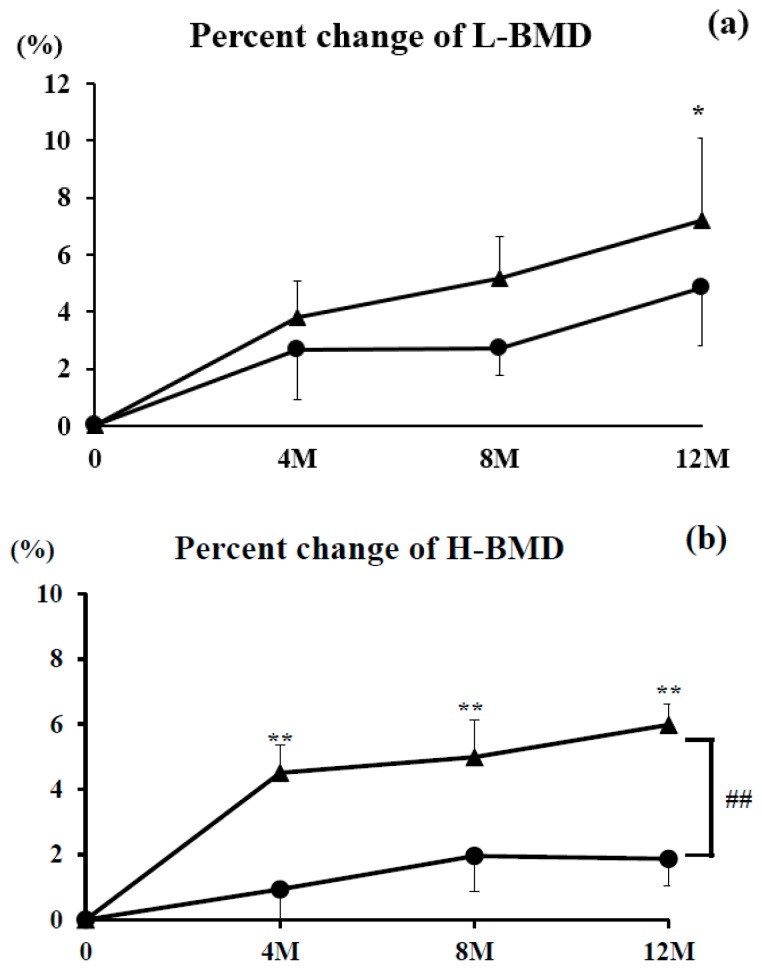
Percent changes in lumbar bone mineral density (L-BMD) (**a**) and bilateral total hip BMD (H-BMD) (**b**) over the 12-month study period. Closed circles show the denosumab monotherapy group and closed triangles show the combination group. Double asterisks (**) denote significant differences (*p* < 0.01) at each time point compared with pretreatment in the denosumab monotherapy or combination groups. Double hashtags (##) show significant differences (*p* < 0.01) between the denosumab monotherapy and combination groups at each time point.

The percent change of L-BMD increased steadily over the study period in the denosumab group (4.8% increase at 12 months) and combination group (7.2% increase at 12 months). There were no significant differences in either group at any time point compared with pre-treatment levels, with the exception of 12 months in the combination group. There were also no significant differences between the groups at any time point (Figure 3a).

The percent change of H-BMD also rose steadily over the observational period in the denosumab group (1.9% increase at 12 months) and combination group (6.0% increase at 12 months). H-BMD was significantly higher in the combination group at every time point (*p* <0.01) compared with pre-treatment levels. There was a significant difference between the groups at 12 months (*p* < 0.01) (Figure 3b).

#### 3.3.4. Indicators of RA State

RA state before treatment was matched in both groups (Table 1). There were no significant differences in the percent changes of MMP-3, DAS28-CRP, SDAI, or HAQ-DI between the groups during follow-up (Table 1 Table 2 and Table 3). 

No serious adverse events, such as hypocalcemia or fracture, were noted during the 12-month study period. 

## 4. Discussion

We report for the first time comparative data between denosumab with or without vitamin D and calcium supplementation in Japanese OP patients with RA. Compared with denosumab monotherapy, combination therapy of denosumab with vitamin D and calcium significantly increased percent changes of H-BMD.

Denosumab is a potent anti-resorption agent. In the Denosumab Fracture Intervention Randomized Placebo Controlled Trial (DIRECT) carried out in Japan, Sugimoto et al. reported that all patients who took daily supplements containing ≥600 mg calcium and 400 IU vitamin D had a significantly decreased risk of vertebral fracture and no hypocalcemia while taking denosumab for 3 years [14]. However, studies focusing on the effectiveness and/or adverse effects of denosumab with or without vitamin D supplementation in OP, particularly with RA, are lacking. Orimo et al. noted that alendronate plus alfacalcidol, a source of active vitamin D, exerted additive effects on the prevention of vertebral fractures and an increase in BMD [17]. Leslie et al. reported that the greater the increase in H-BMD, the lower the fracture risk [18]. These results suggest that increasing BMD reduces the risk of fracture. In this study, H-BMD at 12 months after denosumab therapy was significantly increased in the combination group compared with the denosumab monotherapy group, although no fractures occurred in either group. Thus, in Japan, it may be of benefit to prescribe vitamin D and calcium supplementation for osteoporotic patients with RA to prevent fracture. 

We previously reported that denosumab had strong inhibitory effects on bone resorption from as early as 1 week after administration in primary OP [19]. Here, similar findings were observed in terms of significant decreases in the bone resorption markers TRACP-5b and urinary NTX in OP with RA. The bone formation markers BAP and P1NP decreased mildly in the early stages, but became significantly decreased from 2 months in both groups. Bone resorption and formation usually change in parallel due to the phenomenon of coupling [20], but this was not the case for denosumab. Taken together, the drug appears to have strong inhibitory effects on bone resorption and mild inhibitory effects on bone formation soon after therapy commencement in both primary OP and OP with RA. 

Body et al. reported that denosumab without vitamin D or calcium in the OP patients with breast cancer caused significant hypocalcemia, albeit at double the dose of denosumab (120 mg every 4 weeks) [13]. The results in this study showed no hypocalcemia in the denosumab monotherapy group and no serious adverse effects in either group. The addition of vitamin D and calcium did not markedly decrease serum levels of calcium in the combination group apart from at 1 week. Decreased serum calcium was observed in the denosumab monotherapy group from 1 week to 8 months, especially at 1 week, suggesting that hypocalcemia could be prevented during denosumab treatment by supplementation with vitamin D and calcium.

Whereas denosumab alone had increased L-BMD by 4.8% and H-BMD by 1.9% at 12 months, the combination group achieved L-BMD and H-BMD improvements of 7.2% and 6.0%, respectively. There was a significant increase in the percent change of H-BMD in the combination group at 12 months of treatment and a statistically larger increase of H-BMD between the groups. At present, the mechanism of this enhanced H-BMD gain is unknown, but may be related to the lumbar region being less solicited than the hips; increased H-BMD may be linked to the fact that hip osteoblasts have mechanoreceptors that become more fully activated combined with increased mobility of the analyzed are (i.e., greater in the hips and lower in the lumber area). Also, the reasons why H-BMD in the combination group were significantly greater than those in the denosumab monotherapy group were due to the effects of vitamin D (anti-inflammatory phenomena and bone synthesis) [21]. 

Bellavia et al. have described that vitamin D is a key player in calcium and phosphate homeostasis and an important immunoregulatory molecule [21]. They found that in RA patients, low 25(OH)D levels were significantly associated with clinical parameters of disease activity as well as with high serum levels of IL-17 and IL-23 and bone loss. Accordingly, vitamin D deficiency may play a role in the pathogenesis of osteoporotic patients with RA [21]. Although serum 25(OH)D levels were not measured in this study, vitamin D and calcium supplementation can inhibit the decrease of calcium and improve BMD due to the potential increase of 25(OH)D. Antoniucci et al. reported that vitamin D status at therapy initiation did not affect the BMD response to ALN when co-administered with vitamin D [22]. Bourke et al. described that baseline dietary intake of calcium and vitamin D status did not alter the effects of zoledronate and concluded that co-administration of calcium and vitamin D with zoledronate might be unnecessary for individuals not at risk for marked deficiency of vitamin D [23]. On the other hand, Heckman et al. reported that in elderly patients with OP that was refractory to BPs, vitamin D (1000 IU daily) could improve BMD at the lumbar spine [24]. Roux et al. witnessed that the success of ALN therapy for OP might depend on vitamin D status [25]. Whether vitamin D sufficiency or administration influences the effects on BMD during BP therapy is controversial [22,23,24,25], although it remains generally supported that vitamin D addition is desirable during BP treatment. Reported discrepancies may be due to such population characteristics as age and physical activity and/or genetic factors, such as mutations or polymorphisms in genes of vitamin D [21].

The main limitation of this investigation is its small sample size. A subsequent long-term observational period also will be needed to clarify if: (1) BMD increases continuously by denosumab and to what extent fractures are prevented; and (2) hypocalcemia or adverse effects will later occur.

## 5. Conclusions

Calcium and vitamin D addition to denosumab represents an important treatment option with additive effects on the increase of H-BMD in osteoporotic patients with RA.

## Figures and Tables

**Table 1:** Patient characteristics at baseline. Data are expressed as mean ± standard error.

Characteristic	Denosumab (*n* = 22)	Combination (*n* = 21)	*p*-Value
Age (years)	70.9 ± 1.8	70.6 ± 2.3	0.9161
Gender (F:M)	22:0	21:00	
BMI (kg/m^2^)	20.8 ± 0.9	20.0 ± 0.9	0.5396
Serum corrected Ca (mg/dL)	9.4 ± 0.1	9.2 ± 0.1	0.2156
Serum P (mg/dL)	3.7 ± 0.1	3.5 ± 0.1	0.2378
Serum BAP (μg/L)	13.4 ± 0.6	13.8 ± 1.1	0.7631
Serum TRACP-5b (mU/dL)	302.9 ± 16.7	312.3 ± 29.7	0.7873
Urinary NTX (nmol BCE/mmol/CRE)	28.4 ± 2.5	26.7 ± 1.8	0.6015
1,25(OH)2D3 (pg/mL)	62.8 ± 4.9	60.7 ± 5.7	0.7847
P1NP (μg/L)	37.3 ± 4.2	36.0 ± 4.5	0.8239
Serum whole PTH (pg/dL)	29.7 ± 2.9	30.1 ± 4.2	0.9429
BP use, *n* (%)	22 (100)	21 (100)	
Period of BP use	5.8 ± 1.0	5.5 ± 1.0	0.8476
L-BMD (g/cm^2^)	0.71 ± 0.04	0.68 ± 0.02	0.4197
H-BMD (g/cm^2^)	0.487 ± 0.03	0.502 ± 0.02	0.6971
MTX, *n* (mg/week)	12 (6.7 ± 0.75)	10 (6.6 ± 0.85)	0.9536
PSL, *n* (mg/day)	2 (4.5 ± 0.5)	1 (5.0 ± 0.0)	
MMP-3	92.2 ± 22.2	94.3 ± 15.5	0.9392
Disease duration (years)	16.6 ± 2.9	18.5 ± 3.2	0.6815
DAS28-CRP	3.1 ± 0.3	3.0 ± 0.3	0.9090
SDAI	4.3 ± 1.0	4.7 ± 1.0	0.7987
HAQ-DI	0.44 ± 0.2	0.39 ± 0.2	0.8215

**Table 2:** Value changes at 12 months of treatment. **D**ata are expressed as mean ± standard error.

Characteristic	Denosumab (*n* = 22)	Combination (*n* = 21)	*p*-Value
L-BMD (g/cm^2^)	0.74 ± 0.03	0.72 ± 0.04	0.6983
H-BMD (g/cm^2^)	0.49 ± 0.02	0.54 ± 0.03	0.2236
MMP-3 (ng/mL)	68.6 ± 9.6	56.0 ± 8.6	0.3518
DAS28-CRP	2.7 ± 0.3	2.0 ± 0.3	0.0793
SDAI	4.6 ± 1.1	4.7 ± 1.4	0.9327
HAQ-DI	0.38 ± 0.2	0.28 ± 0.1	0.6359

**Table 3:** Percent changes at 12 months of treatment. **D**ata are expressed as mean ± standard error.

Characteristic	Denosumab (*n* = 22)	Combination (*n* = 21)	*p*-Value
L-BMD (%)	4.8 ± 2.0	7.2 ± 2.8	0.5069
H-BMD	1.9 ± 1.0	6.0 ± 0.6	0.0060
MMP-3 (%)	−4.9 ± 25.6	−13.0 ± 9.7	0.7777
DAS28CRP (%)	−9.7± 6.7	−24.5 ± 10.4	0.2476
SDAI (%)	−7.6 ± 9.7	−31.1 ± 16.9	0.2500
HAQ-DI (%)	−3.6 ± 26.1	−0.5 ± 3.6	0.9095
